# Submucosal Duodenal Artery Pseudoaneurysm Causing Massive Gastrointestinal Hemorrhage: A Case Report

**DOI:** 10.5811/cpcem.2022.3.54564

**Published:** 2022-05-06

**Authors:** Stephen Elliott, Anna Trtchounian, Babak Danesh, Wojciech Bober, David Levy

**Affiliations:** * Good Samaritan Hospital, Department of Emergency Medicine, West Islip, New York; † Good Samaritan Hospital, Department of Gastroenterology, West Islip, New York

**Keywords:** case report, pseudoaneurysm, gastrointestinal hemorrhage, massive transfusion protocol

## Abstract

**Introduction:**

Acute upper gastrointestinal bleeding is a common emergency presentation. The United States Centers for Disease Control and Prevention 2018 survey of emergency department (ED) visits reported 436,000 ED visits for unspecified gastrointestinal bleeding that year.^1^

**Case Report:**

We present the case of a submucosal duodenal pseudoaneurysm causing massive gastro-intestinal hemorrhage in a male on anticoagulation.

**Conclusion:**

Prompt recognition of critical gastrointestinal bleeding, appropriate ED management, and early consultation for emergent intervention are the essential components to reduce morbidity and mortality for patients with massive gastrointestinal hemorrhages.

## INTRODUCTION

Upper gastrointestinal (GI) bleeding has been estimated to account for up to 20,000 deaths annually in the United States.^2^ Overall incidence of acute upper GI hemorrhage has been estimated to be 50–100/100,000 annually.^2^ Upper GI bleeding is defined as bleeding that occurs from a source proximal to the ligament of Treitz. Bleeding from the upper GI tract is about four times more common than bleeding from the lower GI tract.^3^ Common presentations of acute upper GI hemorrhage include hematemesis and/or melena.^4^ The identification and early resuscitation of these patients is pivotal, as the mortality rate of upper GI bleeding can be from 6–10%.^5^ This rate significantly increases and can be fatal when a ruptured pseudoaneurysm is the source of a GI bleed.^6^

## CASE REPORT

An 82-year-old male, with a past medical history significant for atrial fibrillation on apixaban and amiodarone, presented to our emergency department (ED) with coffee-ground emesis, fatigue, and maroon-colored stool. Symptoms started 18 hours prior to arrival. The patient denied abdominal pain, use of non-steroidal anti-inflammatory medications, history of GI bleeding, peptic ulcer disease, or aortic pathology. Other medical history included hyperlipidemia and hypertension for which he was taking metoprolol and amlodipine. All other review of systems was negative.

The patient was an elderly Black male who was diaphoretic, with dry, crusted dark-colored emesis on chin and chest, and with copious foul-smelling, dark-colored stools. The patient’s initial vitals were blood pressure of 86/37 millimeters of mercury (mm Hg), heart rate 60 beats per minute (BPM), respiratory rate 30 breaths per minute, rectal temperature 96° Fahrenheit, and oxygen saturation of 100% on two liters by nasal cannula. The patient had labored breathing, with unremarkable lung sounds on auscultation. His abdomen was soft, non-tender, and non-distended. A point-of-care blood analysis showed an immeasurable “low” hemoglobin grams per deciliter (g/dL) (reference range 12–17g/dL and reportable range 5.1–25.5g/dL) and a hematocrit of less than 15% (reference range 38–51% and reportable range 15–75%).

Two large-bore peripheral intravenous catheters, a central venous catheter, and an arterial monitoring catheter were established. Massive blood transfusion protocol was activated. A total of 10 units of packed red blood cells, four units of fresh frozen plasma, and one unit of platelets were rapidly transfused. Prothrombin complex concentrate, proton pump inhibitor, and vasopressors were administered. The patient was endotracheally intubated for airway protection. After these measures, his blood pressure improved to 112/67 mm Hg with a heart rate in the 50s BPM. Repeat hemoglobin was 7.0 g/dL.

**Image 1:**
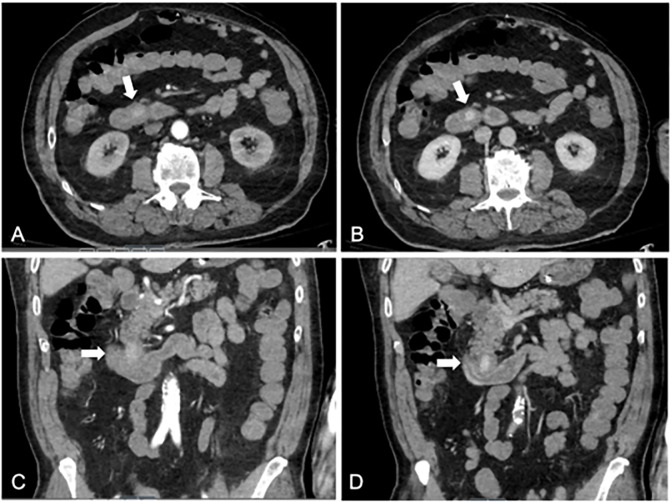
Computed tomography angiogram of abdomen showing submucosal duodenal pseudoaneurysm (white arrows) causing massive gastrointestinal hemorrhage in (A) transverse plane arterial phase, (B) transverse plane venous phase, (C) coronal plane arterial phase, and (D) coronal plane venous phase.

Emergent bedside upper GI endoscopy revealed an area of extrinsic compression on duodenum with a fresh blood clot and minimal oozing. Abdominal computed tomography (CT) angiogram demonstrated a 2.5–centimeter pseudoaneurysm in the anterior aspect of the proximal transverse duodenum (Image 1) with no evidence of aortic involvement. The pseudoaneurysm was deemed to be likely submucosal in location with mild surrounding mucosal edema.

**Image 2:**
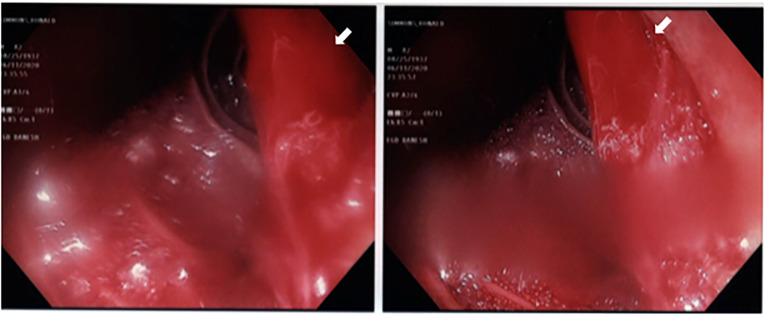
Endoscopy images of active arterial bleeding (white arrow) from submucosal duodenal pseudoaneurysm that caused a massive gastrointestinal hemorrhage.

A repeat bedside upper GI endoscopy showed an intense submucosal hemorrhage with brisk bleeding (Image 2). Endoscopic maneuvers with submucosal epinephrine injection with good blanching and the placement of a hemostatic clip failed to achieve hemostasis. A location to place another clip could not be identified due to intensity of bleeding.

The patient was emergently taken to the operating room for an exploratory laparotomy and retroperitoneal exploration. A pulsatile mass was found within the third portion of the duodenum, abutting the pancreatic head, with active pulsatile hemorrhage. The bleeding vessel was not accurately identified due to the vast amount of bleeding. A partial duodenectomy was performed to remove this mass, which pathology specimen analysis confirmed was a ruptured submucosal pseudoaneurysm within the duodenum. No further GI bleeding was encountered. The patient’s hospital course was complicated by postoperative ileus and Gram-negative septicemia. He gradually improved, was re-started on anticoagulation therapy, and discharged to a rehabilitation facility.

## DISCUSSION

Visceral artery pseudoaneurysms, defined as those affecting the celiac, superior or inferior mesenteric arteries, and/or their branches, are rare with a reported incidence of 0.1–0.2%.^7^ However, the exact number is difficult to account for because most patients are asymptomatic.^7^,^8^ The diagnosis is on the rise due to incidental detection from increased use of advanced imaging techniques and iatrogenic causes from an increase in procedures that use instrumentation.^7^ The most common artery involved is the splenic artery.^7^,^8^

Although the exact vessel could not be identified in this case, based on imaging and surgical findings, a pseudoaneurysm of one of the terminal branches of the pancreaticoduodenal artery of the proximal transverse duodenum was the most likely culprit. Less than 2% of all visceral aneurysms are comprised from pancreaticoduodenal artery.^8^ Pseudoaneurysms have a high incidence of rupturing; thus, patients can present with signs of hemodynamic collapse from catastrophic GI bleed.^7^ Mortality rate of pseudoaneurysm rupture into the GI tract ranges from 25–70%.^7^,^8^

**CPC-EM Capsule**

What do we already know about this clinical entity?

*Acute upper gastrointestinal (GI) bleeding is a common culprit in patients presenting to emergency departments with hematemesis and/or melena*.

What makes this presentation of disease reportable?

*A duodenal arterial pseudoaneurysm is a rare cause of acute upper GI bleeding and is associated with a high mortality rate*.

What is the major learning point?

*Have a high level of suspicion for active GI bleeding to appropriately stabilize and resuscitate these critically ill patients prior to definitive therapy*.

How might this improve emergency medicine practice?

*We review the steps of management in the emergency department for these complex patients and emphasize the importance of getting specialists involved early when minutes matter*.

The acute ED management of GI bleeding is critical. In patients with abnormal vital signs due to active bleeding, initial resuscitation may involve activating massive transfusion protocol based on clinical impression rather than a laboratory value. Current evidence supports transfusion of plasma to platelets to packed red blood cells in either a 1:1:1 or a 1:1:2 ratio.^9^ Platelet transfusion is recommended if the platelet count is less than 50,000 per microliter.^4^ Intubation should be considered in patients who are not protecting their airway or who are at risk of aspiration from ongoing hematemesis. However, intubating a patient with an upper GI bleed has also been associated with increased risk of adverse cardiopulmonary events such as pulmonary edema, acute respiratory distress syndrome, myocardial infarction, or cardiac arrest.^10^

Pharmacotherapy for all patients with suspected severe upper GI bleeding generally include a proton pump inhibitor (PPI). Recommendations include administering a dose equivalent of esomeprazole 80 milligrams (mg) when there is evidence of active bleeding and 40 mg if no evidence of active bleeding. Studies have shown that intermittent PPI is not inferior to continuous PPI.^4^ However, the decision to use a PPI continuous drip should be made in consultation with a gastroenterologist. Somatostatins, such as octreotide, are not recommended in nonvariceal upper GI bleeds.^4^ Patients suspected of or with a history of cirrhosis have demonstrated benefits and decreased mortality from the administration of prophylactic antibiotics.^4^ Tranexamic acid demonstrated no benefit in GI bleeding.^11^

Consideration must be given to reversal of anticoagulation. Recent recommendations include administration of prothrombin complex concentrate and vitamin K to patients on warfarin with elevated international normalized ratio levels. If prothrombin complex concentrate is not available, fresh frozen plasma can be given instead.^4^ Direct oral anticoagulants (DOAC) generally have a lower risk of life-threatening and/or fatal bleeding in comparison to warfarin. Reversal of DOACs can be more challenging as only dabigatran has a specific reversal agent. For patients on DOACs, recommendations include administration of prothrombin complex concentrate or andexanet alfa, which binds to factor Xa inhibitors.^4^

After resuscitation, a computed tomography angiography may be obtained to detect the source of upper GI bleeding. Consultation with a gastroenterologist is essential since the diagnostic modality of choice is endoscopy. Once the bleeding site is located, hemostasis can be obtained endoscopically.^4^ Hemostasis may not be achieved in ruptured pseudoaneurysms, in which case, surgery or an interventional radiology team may attempt to stop the bleed by surgically removing the problem area or by arterial embolization.^8^ Studies have shown that surgical treatment of visceral pseudo-aneurysm is associated with higher mortality in comparison to treatment by embolization.^5^,^8^ Balloon tamponade may be performed in the ED when endoscopic, surgical, or interventional radiology treatment modalities are not rapidly available for patients with life-threatening upper GI hemorrhages.^5^ This procedure is a temporary means of stabilization until more definitive therapy can be obtained.^5^ A crucial step is early consultation with a gastroenterologist as well as with a surgeon and/or interventional radiologist as adjuncts for potential surgical intervention.

## CONCLUSION

Acute upper GI bleeds, although a common emergency presentation, can be life-threatening, especially if the culprit is a ruptured pseudoaneurysm. Close attention must be paid to alarming vital signs and clinical appearance. Given the pivotal role of early and adequate resuscitation, its importance cannot be overemphasized. The triad of prompt recognition of critical upper GI bleeds, appropriate early management, and early consultation for emergent intervention are the essential components to reduce morbidity and mortality in patients with massive GI hemorrhages.
